# Supplementary material for: Using geographically weighted regression analysis to cluster under-nutrition and its predictors among under-five children in Ethiopia: Evidence from demographic and health survey
Source: PLoS One. 2021 May 21;16(5):e0248156. doi: 10.1371/journal.pone.0248156 (PMC8139501; doi:10.1371/journal.pone.0248156)
Supplement: S1 File — (PDF) [file pone.0248156.s001.pdf]

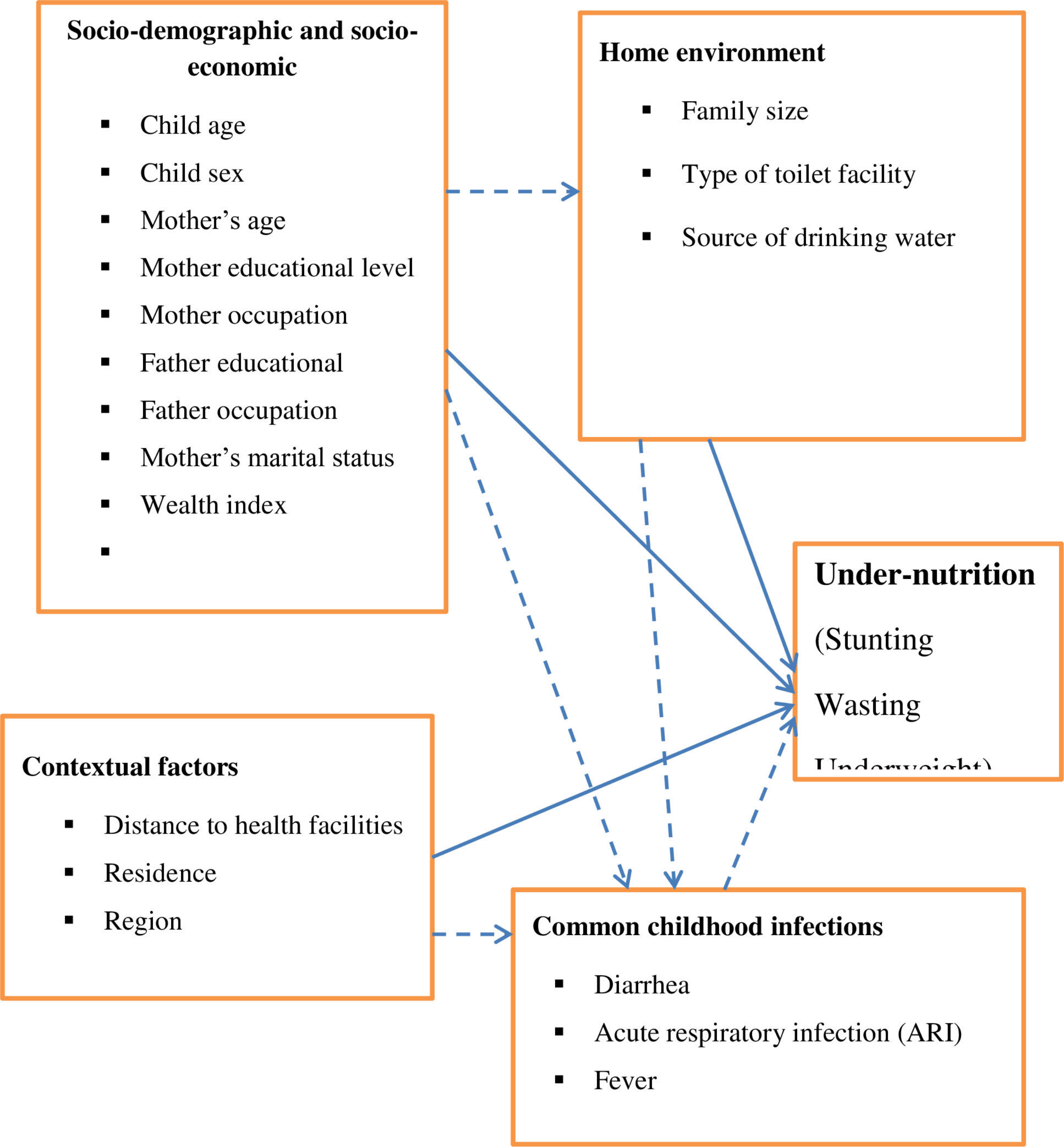
Socio-demographic and socio-economic
Child age
Child sex
Mother’s age
Mother educational level
Mother occupation
Father educational
Father occupation
Mother’s marital status
Wealth index
Home environment
Family size
Type of toilet facility
Source of drinking water
Under-nutrition
(Stunting
Wasting
Contextual factors
Distance to health facilities
Residence
Region
Common childhood infections
Diarrhea
Acute respiratory infection (ARI)
Fever
