# Supplementary material for: Using geographically weighted regression analysis to cluster under-nutrition and its predictors among under-five children in Ethiopia: Evidence from demographic and health survey
Source: PLoS One. 2021 May 21;16(5):e0248156. doi: 10.1371/journal.pone.0248156 (PMC8139501; doi:10.1371/journal.pone.0248156)
Supplement: S2 File — (PDF) [file pone.0248156.s002.pdf]

# Summary of OLS Results - Model Variables

| Variable | Coefficient [a] | StdError | t-Statistic | Probability [b] | Robust_SE | Robust_t | Robust_Pr [b] | VIF [c] |
|---|---|---|---|---|---|---|---|---|
| Intercept | 20.394627 | 3.165809 | 6.442154 | 0.000000* | 3.325245 | 6.133271 | 0.000000* | -------- |
| TUNIMPRPER | 0.186728 | 0.031327 | 5.960623 | 0.000000* | 0.029157 | 6.404246 | 0.000000* | 2.551101 |
| FEMALEPE | 0.133730 | 0.040120 | 3.333258 | 0.000923* | 0.049108 | 2.723196 | 0.006641* | 1.003209 |
| RURALPER | -0.030603 | 0.025462 | -1.201935 | 0.229836 | 0.023946 | -1.278042 | 0.201706 | 3.058432 |
| FPRIMPER | -0.127514 | 0.042162 | -3.024422 | 0.002602* | 0.045384 | -2.809688 | 0.005114* | 1.060039 |
| FSECONP | -0.206403 | 0.030257 | -6.821720 | 0.000000* | 0.031941 | -6.462106 | 0.000000* | 1.875048 |
