# Supplementary material for: Using geographically weighted regression analysis to cluster under-nutrition and its predictors among under-five children in Ethiopia: Evidence from demographic and health survey
Source: PLoS One. 2021 May 21;16(5):e0248156. doi: 10.1371/journal.pone.0248156 (PMC8139501; doi:10.1371/journal.pone.0248156)
Supplement: S3 File — (PDF) [file pone.0248156.s003.pdf]

Summary of OLS Results - Model Variables

Variable	Coefficient [a]	StdError	t-Statistic	Probability [b]	Robust_SE	Robust_t	Robust_Pr [b]	VIF [c]
Intercept	5.377181	1.192933	4.507531	0.000010*	1.469349	3.659568	0.000286*	--------
TUNIMPRPER	0.059517	0.021610	2.754079	0.006054*	0.024124	2.467119	0.013870*	2.513701
V8ABOVEPE	0.085499	0.025643	3.334211	0.000920*	0.029261	2.921933	0.003609*	1.101915
RURALPER	0.005595	0.016279	0.343680	0.731213	0.016253	0.344234	0.730796	2.588772
V3549P	-0.115005	0.036343	-3.164425	0.001641*	0.033823	-3.400178	0.000730*	1.095955
FPRIMPER	-0.071297	0.028713	-2.483067	0.013270*	0.015991	-4.458545	0.000012*	1.017992
