# Supplementary material for: Using geographically weighted regression analysis to cluster under-nutrition and its predictors among under-five children in Ethiopia: Evidence from demographic and health survey
Source: PLoS One. 2021 May 21;16(5):e0248156. doi: 10.1371/journal.pone.0248156 (PMC8139501; doi:10.1371/journal.pone.0248156)
Supplement: S4 File — (PDF) [file pone.0248156.s004.pdf]

Summary of OLS Results - Model Variables

Variable	Coefficient [a]	StdError	t-Statistic	Probability [b]	Robust_SE	Robust_t	Robust_Pr [b]	VIF [c]
Intercept	14.548280	2.391914	6.082276	0.000000*	2.182220	6.666735	0.000000*	--------
TUNIMPRPER	0.134182	0.028850	4.651085	0.000006*	0.025576	5.246356	0.000000*	2.604712
BIGPROPE	0.026273	0.022182	1.184451	0.236677	0.023006	1.142007	0.253878	1.527859
MPRIMPE	-0.094737	0.027849	-3.401833	0.000726*	0.032415	-2.922627	0.003601*	1.062254
RURALPER	0.031254	0.023572	1.325941	0.185344	0.021570	1.448952	0.147858	3.155613
V3549P	-0.158624	0.047398	-3.346669	0.000881*	0.038221	-4.150214	0.000043*	1.083819
FSECONP	-0.100582	0.027461	-3.662685	0.000283*	0.027672	-3.634735	0.000314*	1.859492
