# Supplementary material for: Using geographically weighted regression analysis to cluster under-nutrition and its predictors among under-five children in Ethiopia: Evidence from demographic and health survey
Source: PLoS One. 2021 May 21;16(5):e0248156. doi: 10.1371/journal.pone.0248156 (PMC8139501; doi:10.1371/journal.pone.0248156)
Supplement: Attachment — Submitted filename: PLOS One Reviewer.docx [file pone.0248156.s006.docx]

**Reviewer’s comments**

**Full title:** Geographical clustering of under-nutrition and its predictors among under-five children in Ethiopia: Geographically weighted regression Analysis

**Manuscript number:** PONE-D-20-27277

**Corresponding author:** Mequannent Melaku sharew, Mph in health informatics Wollo University Desse, ETHIOPIA

**Comments and questions**

I appreciate the authors for addressing such highly contextually and culturally affected public health issues. It so concerning that under-nutrition is continued to be a public health problem in Ethiopia. Therefore, evidence regarding the distribution of under-nutrition with its respective determinants is important for decision-making in dealing with its prevention and control program. The paper is well organized and presented. The study presents the findings of original research in the area of public health. And the results have not been published elsewhere. Having adequately performed relevant analyses with sufficient detail; it presents coherent and data-oriented conclusions. The article is presented in an acceptable level English language standard and reporting guideline.

**Additional comments and questions**

The author would respond to some of the following questions and comments.

1. **Title:** “Geographical clustering of under-nutrition and its predictors among under-five children in Ethiopia: Geographically weighted regression Analysis”. Since the data for this study comes from the DHS source; it’s good to indicate it in the title. So, the author would restructure the title as…; “Using Geographically weighted regression Analysis to cluster under-nutrition and its predictors among under-five children in Ethiopia: Evidence from demographic and health survey”.
2. **Abstract: Line 30: Methods:** in the method section of the abstract; the author should indicate that the data comes from DHS. Indicating it is very important as it adds quality to the paper (data from a large population) and attracts the readers at a first impression.
3. **Introduction: page: Line 99-10:** “Geographical variation of under-nutrition can delay control and elimination of malnutrition which is the underline cause of most childhood disease that in turn brings a high proportion of under-five mortality.” This sentence is not clear or it seems incomplete. It would good for the author to rephrase it?
4. **Methods and materials: Study setting: Line 110:** the author would briefly discuss information about study settings or Ethiopia. E.g about the nine regional states of Ethiopia and other relevant information to under-nutrition such as urbanization, economic and agricultural practices, etc.
5. **Discussion: Line 374 and the Implications of the study: Line 510:** It gives more sound meaning if the author could combine discussion points with implications. The author tried to compare the findings of the study with other previous pieces of evidence and discuss discrepancies as well. That’s good. But it would be more informative if the author could also discuss the implications (policy, methodological and practical implications) for each point of discussion and explanations. in so doing, the author would remove the section “**Implications of the study in Line 510”.**
6. Question: It’s clear that the distribution of under-nutrition in Ethiopia was indicated in DHS 2016. And the determinants as well. My question is; what specific value (scientific or methodological) did your study added to the existing body of evidence? Would you please indicate it clearly in your discussions? Keep in mind my comments in #5 above.
