# Supplementary material for: Using geographically weighted regression analysis to cluster under-nutrition and its predictors among under-five children in Ethiopia: Evidence from demographic and health survey
Source: PLoS One. 2021 May 21;16(5):e0248156. doi: 10.1371/journal.pone.0248156 (PMC8139501; doi:10.1371/journal.pone.0248156)
Supplement: Attachment — Submitted filename: Reviewers Comments.docx [file pone.0248156.s008.docx]

| **Reviewers Comments** | 1. Subject to data availability, authors have been considerate and dropped 1872 observation which may potentially introduce selection bias in the process of estimation. Authors should clarify all the technical details and may present attrition bias analysis in the method section. 2. Authors have chosen limited set of explanatory variables in the model. So authors should clarify the motivation behind. As it is well documented that childhood undernutrition caused by multitude of factors ( maternal and home environment variable, complementary and breastfeeding practices and infections and other contextual and socioeconomic variable) . Since author is not using any standard analytical framework. It would be great addition in the manuscript if author can present guiding conceptual framework used for selection of explanatory variables. Otherwise it would be a major limitation in terms of assessing risk factors of undernutrition without controlling for number of potential confounders. 3. Author have used both wealth index as well as few variables which is used to create wealth index in general for example type of toilet facility as an explanatory variable. So eventually there will be a bias. Authors should clarify. 4. Categorization of some of the variables need to be relooked like having 8 children and above and fathers and mother’s education completion. Author should include standard education millstones variables for the parents such as secondary school completion (evident from the previous literature in LMIC). Similarly, other combination of children ever born variables (e.g 4 or more) should be modeled to see the results. 5. Authors must provide the details of variation inflation factors (VIFs) in the appendix. To see the multicollinearity pattern. The data consistency should be checked properly. 6. In reference section, wherever possible author should attach DOI. Authors should cite carefully. 7. Author alco can strengthen the discussion section by including the debate on the employed geostatistical tool. It would be beneficial for the readers if author can include the discourse on advantages of using GWR over other existing spatial regression method (spatial lag and error models) |
| --- | --- |
