# Supplementary material for: Using geographically weighted regression analysis to cluster under-nutrition and its predictors among under-five children in Ethiopia: Evidence from demographic and health survey
Source: PLoS One. 2021 May 21;16(5):e0248156. doi: 10.1371/journal.pone.0248156 (PMC8139501; doi:10.1371/journal.pone.0248156)
Supplement: Attachment — Submitted filename: Rebuttal letter final undernutrition.docx [file pone.0248156.s009.docx]

Rebuttal letter	Date: 18 December 2020

**PONE-D-20- 27277**

**Geographical clustering of under-nutrition and its predictors among under-five children in Ethiopia: Geographically weighted regression Analysis**

**Mequannent sharew**

**PLOS ONE**

Dear all,

We would like to thanks for these constructive, building and improvable comments on this manuscript that would improve substance and content of the manuscript. We considered each comments and clarification questions of editors and reviewers on the manuscript thoroughly. Our point-by-point responses for each comment and questions are described in detailed on the following pages. Further, the details of changes were shown by track changes in the supplementary document attached.

**Editor comments**

1.

2.

3.

4.

**Reviewer # 1 comments**

1. Title: “Geographical clustering of under-nutrition and its predictors among under-five children in Ethiopia: Geographically weighted regression Analysis”. Since the data for this study comes from the DHS source; it’s good to indicate it in the title. So, the author would restructure the title as…; “Using Geographically weighted regression Analysis to cluster under-nutrition and its predictors among under-five children in Ethiopia: Evidence from demographic and health survey.

**Response**: Thank you reviewer for the constructive comment to improve our work for further readability and we restructured the tile in the revised manuscript file as: “Using Geographically weighted regression Analysis to cluster under-nutrition and its predictors among under-five children in Ethiopia: Evidence from demographic and health survey”

2. Abstract: Line 30: Methods: in the method section of the abstract; the author should indicate that the data comes from DHS. Indicating it is very important as it adds quality to the paper (data from a large population) and attracts the readers at a first impression.

**Response**: Thank you reviewer for the constructive comment to improve our work for further readability and we indicated that the data comes from the Ethiopia 2016 DHS data in the revised manuscript file shown by highlighting as “The data were obtained from the Ethiopian Demographic and Health Survey (EDHS) 2016”

3. Introduction: page: Line 99-10: “Geographical variation of under-nutrition can delay control and elimination of malnutrition which is the underline cause of most childhood disease that in turn brings a high proportion of under-five mortality.” This sentence is not clear or it seems incomplete. It would good for the author to rephrase it?

**Response**: Thank you reviewer for the constructive comment to improve our work. The statement is not relevant placed in an inappropriate flow and we removed it in the revised manuscript file.

4. Methods and materials: Study setting: Line 110: the author would briefly discuss information about study settings or Ethiopia. E.g. About the nine regional states of Ethiopia and other relevant information to under-nutrition such as urbanization, economic and agricultural practices, etc.

**Response**: Thank you reviewer for the constructive comment to improve our work. We have included statement which describe About the nine regional states, urbanization, economic and agricultural practices of Ethiopia in the manuscript.

5. Discussion: Line 374 and the Implications of the study: Line 510: It gives more sound meaning if the author could combine discussion points with implications. The author tried to compare the findings of the study with other previous pieces of evidence and discuss discrepancies as well. That’s good. But it would be more informative if the author could also discuss the implications (policy, methodological and practical implications) for each point of discussion and explanations. In doing so, the author would remove the section “Implications of the study in Line 510”.

**Response**: Thank you very much reviewer for real supportive suggestions provided to enhance the scientific contribution and implications of our work. In this regard I really appreciate over all the issues raised and we tried to address them point by point.

We removed the sub heading Implications of the study and merged the ideas with the discussion at the end.

6. Question: It’s clear that the distribution of under-nutrition in Ethiopia was indicated in DHS 2016. And the determinants as well. My question is; what specific value (scientific or methodological) did your study added to the existing body of evidence? Would you please indicate it clearly in your discussions? Keep in mind my comments in #5 above.

**Response**: Thank you reviewer for the question for clarity. Even though we are using data from the 2016 Ethiopia DHS the current study are different from the DHS report:

The current study used spatial analysis techniques i.e. Geographic weighted regression models to explore the geographical variation of under-nutrition and its predictors among under-five children at zonal levels of Ethiopia but the DHS simply present the finding mainly the prevalence of under-nutrition using descriptive statistics mainly frequency and percentage. In addition the DHS simply describe the characteristics of under-five children not show their association with the outcome variable since they didn’t report the predictors of under-nutrition using analytical models but the current study identified the predictors of under-nutrition at zonal level of Ethiopia.

Furthermore the current study identified the high risk hot spot zones for under-nutrition using spatial hot spot analysis and showed significant clusters for under-nutrition by using spatial sat scan software with Bernouli model But the DHS didn’t address these issues and / lacks.

**Reviewer # 2 comments**

1. Subject to data availability, authors have been considerate and dropped 1872 observation which may potentially introduce selection bias in the process of estimation. Authors should clarify all the technical details and may present attrition bias analysis in the method section.

**Response**: thank you reviewer for the supportive comments. A total of 10,640 under-five children were included in the 2016 EDHS survey and eligible for Height and weight (Anthropometric) measurements. From this 1872 under-five children were dropped since valid or complete data were not obtained during data collection. That is why we dropped them from the analysis.

2. Authors have chosen limited set of explanatory variables in the model. So authors should clarify the motivation behind. As it is well documented that childhood under-nutrition caused by multitude of factors (maternal and home environment variable, complementary and breastfeeding practices and infections and other contextual and socio-economic variable). Since author is not using any standard analytical framework. It would be great addition in the manuscript if author can present guiding conceptual framework used for selection of explanatory variables. Otherwise it would be a major limitation in terms of assessing risk factors of under-nutrition without controlling for number of potential confounders.

**Response**: Thank you very much reviewer for the constructive comments. We provided the guiding conceptual framework of the study in the revised manuscript file Adopted from a study by Mengistu K, Alemu K, Destaw B (2013) Prevalence of Malnutrition and Associated Factors Among Children Aged 6-59 Months at Hidabu Abote District, North Shewa, Oromia Regional State. J Nutr Disorders Ther T1: 001 in the revised manuscript file as supportive information (S1).

We also included maternal (Age of the mother, Mother’s educational level, Mother’s employment status) home environment variables (family size, type of toilet facility, source of drinking water) and context and socio-economic variables (wealth index, region, distance to health facility in the manuscript file.

But complementary and Breast-feeding related variables are not included in the analysis since we these variables are not collected for under-two years age children but not under-five children that is why we didn’t incorporate them in our study.

In addition, we have included it as limitation in terms investigating all potential predictor variables due to the nature of the data.

3. Author has used both wealth indexes as well as few variables which are used to create wealth index in general for example type of toilet facility as an explanatory variable. So eventually there will be a bias. Authors should clarify.

**Response**: Thank you reviewer for the comment and we are not computing wealth index rather we took from the DHS report directly to look its impact on childhood under- nutrition as a socio-economic variable. We took toilet facility as home environmental variable to look it’s on childhood under-nutrition since it has scientific association with our outcome of interest. We also conducted multi-variable logistic regression analysis to control/handle confounding effect and toilet facility was not confounder variables rather it is a real predictor of under-nutrition. Wealth index was not found significant in the multi variable regression analysis it is due to confounding effect.

4. Categorization of some of the variables need to be relooked like having 8 children and above and fathers and mother’s education completion. Author should include standard education millstones variables for the parents such as secondary school completion (evident from the previous literature in LMIC). Similarly, other combination of **children ever born** variables (e.g 4 or more) should be modeled to see the results.

**Response**: Thank you very much reviewer for the constructive comments. First, we kindly apologize to labeling the variable having children 8 and above. The variable we have used was having family size 8 and above. Categorization of variables like education and was based on different literatures (reference no 33, 34, 35 and 36)

5. Authors must provide the details of variation inflation factors (VIFs) in the appendix. To see the multi collinearity pattern. The data consistency should be checked properly.

**Response**: Thank you reviewer for the constructive comments and we have included it. Variance inflation factor has been determined for each variable in table 4, table 6 and table 8 on the 5^th^ column. variables which have a variance inflation factor of 7.5 and above were dropped from the model due to multi-collinearity problem. Therefore, variables which has a variance inflation factor of less than 7.5 has been included in the final model. For this reason, we have included as supplementary information (S2, S3, and S4).

6. In reference section, wherever possible author should attach DOI. Authors should cite carefully??/

**Response**: Thank you reviewer for the constructive comments and we evaluated our references consciously and carefully. we have also provided DOI number when possible.

7. Author also can strengthen the discussion section by including the debate on the employed geostatistical tool. It would be beneficial for the readers if author can include the discourse on advantages of using GWR over other existing spatial regression method (spatial lag and error models)

**Response**: Thank you reviewer for the constructive comments and we have included it. This study had used geographically weighted regression analysis that could enables to determine local coefficients a step advance from ordinary least square analysis. In addition, geographically weighted regression analysis improves model performance by employing a spatial weighted function and addresses spatial heterogeneity and also has an advantage of having less biased predicted coefficients compared to spatial lag and spatial error models. Even though spatial lag and spatial error models enable to addresses both spatial heterogeneity and spatial homogeneity, spatial heterogeneity and spatial homogeneity are unique, not mutually exclusive, features of spatial data. Therefore, this model might bring biased coefficients.

**Reviewer # 3 comments**

1. First paragraph line 25: Problem on malnutrition not well described. Indicate the problem in short way of description.

**Response**: Thank you very much reviewer for the constructive comment. We described well the problem malnutrition in the revised manuscript file shown by highlighting “Malnutrition among under-5 year children is a common public health problem and it is one of the main reasons for the death of children in developing countries including Ethiopia.”

2. First paragraph line 28: In the objective you indicated determinants. Do you think this is in line with the title?

**Response**: Thank you reviewer for the constructive comment and we changed determinants to predictors in the revised manuscript file to make consistent with title.

3. Line 36 says statistical decision was made at p value<0.05. What is your confidence interval?

**Response**: Thank you reviewer for the constructive comments and we included confidence level in the revised manuscript file as “a statistical decision was made at p-value<0.05 with 95% CI.

4. Line 46 you listed predictors. But level of significance not outlined. So see and revise appropriate reporting on the abstract so that reader will capture all relevant data from abstract

**Response**: Thank you reviewer for the constructive comments and we included level of significance at P <0.001 in the revised manuscript shown by highlighting.

5. Line 55 One of recommendations you says “geographic based interventions.” So, there are many geographic based interventions and specify to the study findings you outlined in the results.

**Response**: Thank you very much Reviewer for providing constructive and supportive comments to improve the readability of this study. The objective of our study is to explore the spatial variation of under-nutrition at zonal level in Ethiopia using GWR analysis techniques. Thus, geographic based nutritional interventions could be held to reduce the burden of childhood under-nutrition in hot spot areas by mobilizing additional resources. In addition improving sanitation and hygiene practice, improving the life style of the community and promotion of parent education in the identified hot spot zones for under-nutrition should be more emphasized.

6. Line 125: What were your inclusion criteria and exclusion criteria to identify geographical clustering of the area.

**Response**: Thank you reviewer for the constructive comments. There are 645 clusters included in the 2016 EDHS. Of these 23 clusters had no latitude and longitude coordinates. In this study we excluded those clusters had no latitude and longitude coordinates (i.e. spatial or location data) and we included 622 clusters had latitude and longitude coordinates (i.e. spatial or location data).

7. Line 133: how you sampled based on proportional to each cluster size and independent to each sampling stratum? Indicate on the method section. Number of study population is not clearly articulated and indicate it.

**Response**: Thank you very much reviewer for the comments. We described sampling issues clearly in the method section of the revised manuscript file. Also described number of study population. To select study participants a stratified, two-stage cluster sampling technique was employed for the 2016 EDHS. Enumeration areas (EAs) were the sampling units for the first stage and households were the sampling unit for the second stage. In the 2016 EDHS, a total of 645 EAs (202 urban and 443 rural) were selected with a probability proportional to EAs size (based on the 2007 housing and population census) and independent selection in each sampling stratum. Of these, 18,008 households and 16,583 eligible women were included. Additionally, we described the sampling techniques clearly in the figure 2

8. Line 154: “Improved toilet facility: is the toilet facility when it is used only by members of the household” Do you think this is right definition. Review the definition

**Response**; thank you reviewer for the comment and we revised the definition in the revised manuscript file “Improved toilet facility is the toilet facility includes any non-shared toilet of those types: flush/pour flush toilets to piped water systems, septic tanks and, and pit latrines; ventilated improved pit (VIP) latrines, pit latrines with slabs, and composting toilets”

9. Line 176: Good and self- explanatory analysis plan. But try to shorten and use key analysis plan from those written in the document

Response: thank you reviewer for the comment. We have shortened it as far the document could reveal the concept.

10. Line 207: How you calibrated spatial regression model?

Response: thank you reviewer for the comment. Model calibration (model fitness statics) was assessed by using the six checks for a model undergone for geographically weighted regression analysis. Accordingly, the first check was 1. do coefficients has the expected sign (whether positive or negative) 2. Does the model have no redundant variable? 3. does the model has acceptable adjusted R^2?^ 4. Does the p-value significant yes? 5. Does the model has normally distributed residuals yes? 6. Does the model has Spatially uncorrelated residuals yes? This all recommendation has been checked in ordinary least square analysis and summarized accordingly. After all, geographically weighted regression analysis was executed and assessed for model calibration using Aic and ANOVA whether the model improved or not.

11. Line 232: “A total of 9384 under-5 children were included in this study.” What was total number of children included in the EDHS?

**Response**: Thank you reviewer for the comment. A total of 10,640 under-five children were included in the 2016 EDHS survey. Among this valid or complete data were not obtained for some children during data collection.

12. Line 234: Improve reporting of results like “Regarding maternal education, the majority about, 6183 (65.9%)” Just write number and %

**Response**: Thank you reviewer for the supportive comments. We amended it in the revised manuscript file by reporting number and % shown by highlighting “About 6183 (65.9%) of mothers had no formal education.

13. Line 272: How you analyzed Hotspot analysis. Indicate it.

**Response**: Thank you very much reviewer for the constructive comments. We analyzed hot spot analysis using Gettis-Ord Gi* statistics. GIZ-score is computed to determine the statistical significance of clustering, and the *p*-value computed for the significance. If the z-score is between −1.96 and +1.96, the p-value would be larger than 0.05, and could not reject the null hypothesis; the pattern exhibited could very likely be the result of random spatial processes. If the z-score falls outside the range, the observed spatial pattern was probably too unusual to be the result of random chance, and the p-value would be small to reflect this. In this case, it is possible to reject the null hypothesis and proceed with figuring out what might be causing the statistically significant spatial pattern in the data. Statistical output with high Gi* indicates “hotspot” whereas low Gi* means a “cold spot”.

Dark red colors show significant (p-value < 0.001) clusters of Under-nutrition (risk areas), whereas, dark blue colors show significant (p-value < 0.001) non-risk areas. The more clustered red and blue colors indicate more Under-nutrition risk and non-risk areas, respectively. When the Z-score increases (+/−), its significance level increases as shown in figure **7**

14. Line 538: The recommendation is not well written. Recommend based on the results and try to be specific???

**Response**: thank you very much reviewer for the comment to strengthen our work. We amended the recommendation based on our finding in the revised manuscript section shown by highlighting “Thus, geographic based nutritional interventions mainly mobilizing additional resources could be held to reduce the burden childhood under-nutrition in hot spot areas. In addition, improving sanitation and hygiene practice, improving the life style of the community, and promotion of parent education in the identified hot spot zones for under-nutrition should be more emphasized.”

**Reviewer # 4 comments**

1. In the abstract part line 31, says that a stratified two stage cluster sampling was used for to include clusters. However, the stratification and stages of sampling has not available in the manuscript. So it is better you will add it in your manuscript.

**Response**: Thank you reviewer for the constructive and supportive comment to improve our work for readability. We described the stratification and stage of sampling in the revised manuscript file shown by highlighting “Enumeration areas (EAs) were the sampling units for the first stage and households were the sampling unit for the second stage. In the 2016 EDHS, a total of 645 EAs (202 urban and 443 rural) were selected with a probability proportional to EAs size (based on the 2007 housing and population census) and independent selection in each sampling stratum. Of these, 18,008 households and 16,583 eligible women were included. The detailed sampling procedure was presented in the full EDHS report”

2. Your manuscript has no page numbers. You have to add it.

**Response**: thank you very much reviewer for the constructive comments and we provided page number for the revised manuscript file.

| **Editor questions/comments** | **Response** |
| --- | --- |
| 1. Please ensure that your manuscript meets PLOS ONE's style requirements, including those for file naming. The PLOS ONE style templates can be found at | Thank you, dear editor, for the comment. We have written the manuscript according to PLOSE ONE format using the guideline. |
| 2. We suggest you thoroughly copyedit your manuscript for language usage, spelling, and grammar. If you do not know anyone who can help you do this, you may wish to consider employing a professional scientific editing service. | Thanks, reviewer, for your constructive comment. We have revised the language usage, spelling, and grammar of the manuscript. In the track change part of the manuscript, we have highlighted in yellow color the change with regard reviewer’s comment. In addition, language improvement is highlighted with red color. |
| 3. We note that Figures 5, 7, 8, 9, 10, 11 and 12 in your submission contain map images which may be copyrighted. | Thanks, editor, for your comment. All the figures you noted (5, 7, 8, 9, 10, 11 and 12) was produced using shape file from open Africa website (<https://africaopendata.org/dataset/ethiopia-shapefiles>) which freely available website and enumeration area shape file from DHS website(<https://dhsprogram.com/data/dataset_admin/login_main.cfm?CFID=1242368&CFTOKEN=c892a8da9855f981-8D71EDAA-BF68-5950-1D6BA7F0BB37D5E8>) and the we have produced the figures by ArcGIS version 10.7 software. Permission letter to use the Ethiopian enumeration area shape file and non-spatial data is attached as supplementary information(S5). For this reason, we have incorporated the source of Ethiopia shape file URL in each figure. |
| 4. Your ethics statement should only appear in the Methods section of your manuscript. If your ethics statement is written in any section besides the Methods, please delete it from any other section. | Thanks, editor, for your comment. We have removed the ethics statement that found in the declaration section. |
