# Supplementary material for: Using geographically weighted regression analysis to cluster under-nutrition and its predictors among under-five children in Ethiopia: Evidence from demographic and health survey
Source: PLoS One. 2021 May 21;16(5):e0248156. doi: 10.1371/journal.pone.0248156 (PMC8139501; doi:10.1371/journal.pone.0248156)
Supplement: Attachment — Submitted filename: REVIEWER COMMENTS ON GEOGRAPHICAL REGRESSION ON NUTRITION ETHIOPIA.docx [file pone.0248156.s007.docx]

Plos one: Geographical clustering of under-nutrition and its predictors among under-five children in Ethiopia: Geographically weighted regression Analysis

**Manuscript Number: PONE-D-20-27277**

Corresponding Author: Mequannent Melaku sharew, Mph in health informatics Wollo University Desse, ETHIOPIA

Comments:

1. First paragraph line 25 – Problem on malnutrition not well described. Indicate the problem in short way of description
2. First paragraph line 28 – In the objective you indicated determinants . Do you think this is in line with the title?
3. Line 36 says statistical decision was made at p value<0.05. What is your confidence interval?
4. Line 46 you listed predictors. But level of significance not outlined. So see and revise appropriate reporting on the abstract so that reader will capture all relevant data from abstract
5. Line 55 One of recommendations you says “geographic based interventions.” So, there are many geographic based interventions and specify to the study findings you outlined in the results.
6. Line 125 : What was your inclusion criteria and exclusion criteria to identify geographical clustering of the area
7. Line 133: how you sampled based on proportional to each cluster size and independent to each sampling stratum? Indicate on the method section. Number of study population is not clearly articulated and indicate it
8. Line 154: “Improved toilet facility: is the toilet facility when it is used only by members of the household” Do you think this is right definition. Review the definition
9. Line 176: Good and self explanatory analysis plan. But try to shorten and use key analysis plan from those written in the document
10. Line 207: How you calibrated special regression model?
11. Line 232: “A total of 9384 under-5 children were included in this study.” What was total number of children included in the EDHS?
12. Line 234: Improve reporting of results like “Regarding maternal education, the majority about, 6183 (65.9%)” Just write number and %
13. Line 272: How you analyzed Hotspot analysis. Indicate it
14. Line 374: Good discussion
15. Line 538: The recommendation is not well written. Recommend based on the results and try to be specific
